# Tarlatamab in Previously Treated Small Cell Lung Cancer: A Real-World Experience in a Predominantly Hispanic Population with CNS Metastases

**DOI:** 10.3390/cancers18111806

**Published:** 2026-06-01

**Authors:** Santiago Sucre, Chinmay Jani, Dan Morgenstern-Kaplan, Zuniga Nelsy, Rakhi Modak, Kyle Edwards, Brandon Rose, Subul Malik, Kyle Rowley, Asad Rauf, Gilberto Lopes, Estelamari Rodriguez, Aman Chauhan

**Affiliations:** 1 Department of Medicine, University of Miami/Jackson Memorial Hospital, Miami, FL 33136, USA; 2 Sylvester Comprehensive Cancer Center, University of Miami, Miami, FL 33136, USA

**Keywords:** Tarlatamab, small cell lung cancer, real-world evidence

## Abstract

Background: SCLC remains an aggressive malignancy with limited therapeutic options after progression. Tarlatamab, a DLL3-directed bispecific T-cell engager, has a demonstrated survival benefit in clinical trials, but real-world data remain limited. We evaluated the safety, radiographic response, and treatment durability of Tarlatamab in a real-world cohort treated at a single academic center. Methods: We performed a retrospective review of patients with extensive-stage SCLC who received Tarlatamab at the University of Miami Sylvester Comprehensive Cancer Center between 2024 and 31 October 2025. Demographic, clinical, radiographic, and toxicity data were abstracted from electronic medical records. Radiographic response was defined as partial response or stable disease on follow-up imaging. PFS and overall survival OS were estimated using the Kaplan–Meier method. Results: Twenty-three patients were included (median age 72 years), of whom 61% were Hispanic and 61% had brain metastases prior to treatment. Forty-three percent had received two or more prior lines of therapy. CRS and ICANS occurred primarily during Cycle 1 and were limited to grade 1–2 events, with no grade ≥3 toxicities. Median time on treatment was 92 days with a median of four cycles. Among 18 evaluable patients, partial response was observed in 27.7% and stable disease in 16.7%, yielding a disease control rate of 44.4%. Median PFS was 139 days and median OS was 323 days (95% CI, 31–614). Conclusions: In a predominantly Hispanic, heavily pre-treated real-world population with high CNS disease burden, Tarlatamab demonstrated feasible administration, manageable immune-mediated toxicity, and clinically meaningful antitumor activity.

## Simple Summary

This study investigates the real-world use of Tarlatamab, a novel bispecific T-cell engager, in patients with small cell lung cancer (SCLC) who previously received treatment. Conducted at a single academic center in Miami, FL, the study focuses on a predominantly Hispanic population—a group often underrepresented in clinical trials. Our study assessed patient response to Tarlatamab, characterized its safety profile, and examined the durability of treatment effects. Findings revealed that the drug was generally well-tolerated, with manageable side effects, and showed signs of clinical benefit in a subset of patients, including those with brain metastases. By evaluating Tarlatamab in a real-world, diverse setting, this research helps bridge the gap between clinical trial outcomes and everyday clinical practice, offering insights that may guide more personalized treatment decisions for SCLC patients.

## 1. Introduction

Small cell lung cancer (SCLC) accounts for approximately 11–13% of newly diagnosed lung cancers worldwide [1,2]. Despite major therapeutic advances in thoracic oncology, SCLC remains the most aggressive lung cancer subtype, with poor long-term outcomes. Five-year overall survival (OS) rates range from 12 to 30% in limited-stage disease (LS-SCLC) and remain below 15% in extensive-stage disease (ES-SCLC) [3,4,5,6,7]. At initial diagnosis, nearly 70% of patients present with extensive-stage disease, and even with treatment, median OS is only 12–13 months [4,8]. Platinum-based chemotherapy, using cisplatin or carboplatin in combination with etoposide, continues to serve as the treatment backbone for both limited- and extensive-stage SCLC [9]. In the United States alone, approximately 30,000 new cases of SCLC were expected to be diagnosed in 2025 [10].

For LS-SCLC, the CONVERT and CALGB 30610 trials continue to serve as the key landmark studies, establishing that concurrent platinum-based chemoradiation leads to improved survival and superior local control [11,12]. The recent phase 3 ADRIATIC trial demonstrated that the addition of PD-L1 inhibitor durvalumab as consolidation therapy after chemoradiation showed improved progression-free survival (PFS) and OS compared to prior reference populations [13]. For ES-SCLC, first-line therapy consists of platinum plus etoposide in combination with durvalumab or atezolizumab, followed by maintenance immune checkpoint therapy [9]. This approach became the standard of care after the IMpower133 and CASPIAN trials demonstrated improved median OS (mOS) with the addition of PD-L1 inhibitors to initial chemoimmunotherapy [14,15]. More recently, the IMforte trial demonstrated improved PFS and OS with the addition of lurbinectedin to atezolizumab as first-line maintenance therapy in patients without disease progression after induction treatment [16]. Radiation therapy is reserved for targeted control of metastatic sites and for the alleviation of symptoms [9].

Management in the second-line setting remains challenging and continues to evolve. Historically, systemic therapy options include Topotecan, lurbinectedin, and platinum rechallenge [9], although impact in survival beyond the first-line setting remains modest [17,18,19,20,21,22,23,24]. The DeLLphi-301 and DeLLphi-304 trials showed that Tarlatamab, a bispecific T-cell engager that binds to both DLL3 in cancer cells and CD3 in T-cells [25,26], significantly improved OS compared to chemotherapy in patients with SCLC that progressed during or after platinum-based therapy (13.6 months vs. 8.3 months) [27,28], expanding therapeutic options and establishing Tarlatamab as the current preferred second-line agent [9].

Real-world experience with Tarlatamab continues to expand [29,30], and our study aims to add to the growing evidence by describing its use at a single academic center in Miami with a large Hispanic patient population, focusing on radiographic response and treatment durability.

## 2. Materials and Methods

In this retrospective single-institutional study, we reviewed all patients who received Tarlatamab at the University of Miami Sylvester Comprehensive Cancer Center between 2024 and 31 October 2025. All eligible patients treated during this period were included consecutively, and no additional selection criteria were applied. ES-SCLC patients who had progressed on at least one prior line of therapy were included. Demographic data, treatment details, number of prior therapy lines, and clinical outcomes were collected through electronic medical record review. Radiographic information including baseline CNS involvement at initial diagnosis and during treatment course was also captured. Tarlatamab was administered per the FDA-approved package insert, and Cycle 1 (C1) was given in a monitored inpatient setting in accordance with standard-of-care practices.

Continuous variables were expressed as means with standard deviations or medians with interquartile ranges (IQRs). Categorical variables were summarized as frequencies and percentages. Overall best radiographic response was reported as per RECIST criteria. Overall response rate (ORR) and disease control rate (DCR) were calculated.

Survival analysis was performed in our patient cohort to assess treatment outcomes. Initially, a univariate analysis was conducted, followed by a multivariable analysis performed in an exploratory manner due to the small sample size. Survival outcomes were estimated using the Cox-regression analysis and Kaplan–Meier Survival curves were created.

## 3. Results

### 3.1. Patient Baseline Characteristics

A total of 23 patients were included in this observational cohort. Patient characteristics are summarized in Table 1. The median age was 72 (range from 56 to 87). Most patients were male (61%) and identified as Hispanic (61%). All patients had private insurance coverage.

All patients had a history of smoking; former smokers comprised the majority of the cohort (83%), while four patients were current smokers (17%). ECOG performance status was 0 in 3 patients (13%), 1 in 19 patients (83%), and 2 in 1 patient (4%). Patients had a high comorbidity burden, with a mean Charlson Comorbidity Index of 9.0 ± 2.1. At the time of initial diagnosis of SCLC, 10 patients had LS-SCLC (43%) and 13 patients had ES-SCLC (57%). Brain metastases were present at diagnosis in three patients (13%). Prior to initiation of Tarlatamab, 14 patients (61%) had evidence of brain metastases on baseline imaging obtained within 8 weeks of treatment initiation, including new, stable, or progressive lesions. Among those 14 patients with brain metastases, 8 (57.1%) had previously received stereotactic radiosurgery (SRS), 4 (28.6%) had received whole-brain radiation therapy (WBRT), and 2 (14.3%) had not received prior local therapy. None of the evaluable patients experienced CNS progression while receiving Tarlatamab during the observation period. Liver metastases at diagnosis were observed in three patients (13%), and adrenal metastases were present in one patient (4%) at diagnosis.

In terms of prior treatment exposure, patients had received a mean of 1.7 ± 0.9 prior lines of therapy; 13 patients (57%) received one prior line, 5 patients (22%) received two lines, 4 patients (17%) received three lines, and 1 patient (4%) received four prior lines. Therapies immediately prior to Tarlatamab were heterogeneous, most commonly consisting of platinum-based chemoimmunotherapy, with a subset of patients receiving later-line agents such as lurbinectedin or immunotherapy-based regimens. Immune checkpoint inhibitor therapy as the immediate prior line was given to 16 patients of the cohort (70%).

### 3.2. Immune-Mediated Toxicity Profile

The tolerability profile of Tarlatamab was characterized primarily by Cytokine Release Syndrome (CRS) and Immune Effector Cell-Associated Neurotoxicity Syndrome (ICANS). Adverse events are summarized in Table 2.

On Cycle 1 Day 1 (C1D1), CRS occurred in 4 patients (17%): two grade 1 events and two grade 2 events. On Cycle 1 Day 8 (C1D8), CRS was observed in three patients (13%), with 2 grade 1 cases and one grade 2 episode. No grade 3 or higher CRS were reported at either timepoint in our cohort. Among those who developed CRS, the median time to onset was 7.5 h (IQR, 5.8–11) on Day 1 after Tarlatamab infusion. On Day 8, the median time to onset was 17 h (IQR, 11.5–44.5). The median time to resolution was 8.5 h (IQR, 1.8–27.8) on Day 1 and 7 h (IQR, 6–7) on Day 8. Dexamethasone was administered for CRS management in three out of four cases on Day 1 (75%) and in two out of three cases on Day 8 (67%).

**Figure 1:**
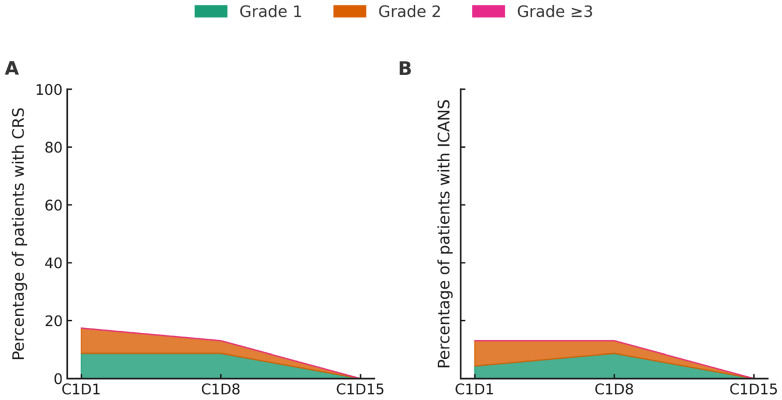
Graphical representation of frequency of immune-mediated adverse events during the first cycle of Tarlatamab: (**A**) Cytokine Release Syndrome frequency on Cycle 1 Day 1, Cycle 1 Day 8, and Cycle 1 Day 15; (**B**) Immune Effector Cell-Associated Neurotoxicity Syndrome frequency on Cycle 1 Day 1, Cycle 1 Day 8, and Cycle 1 Day 15.

Similarly, incidence of ICANS was limited and predominantly low in grade. On both C1D1 and C1D8, ICANS occurred in three patients (13%). On C1D1, one event was grade 1 and two events were grade 2. On C1D8, two events were grade 1 and one event was grade 2. No grade 3 or higher neurotoxicity were observed. Dexamethasone was used for ICANS in two of the three affected patients at each timepoint (67%). There were no CRS or ICANS after C1D15 in our cohort. In three patients, Tarlatamab was stopped due to toxicity. Figure 1 summarizes the frequency and severity profiles of CRS and ICANS.

### 3.3. Time on Tarlatamab and Disease Outcomes

Among the 23 patients in our cohort treated with Tarlatamab, the median time on treatment was 92 days (IQR, 33–192.5), and the median number of treatment cycles administered was four (IQR, 1.5–7.5). Radiographic response was evaluable in 18 patients, five patients were not evaluable as two died after C1D8 admission, one transferred care to another facility, and two were lost to follow-up.

Among the 18 patients with evaluable disease, a radiographic partial response (PR) was observed in 27.7% (*n* = 5), and stable disease (SD) in 16.7% (*n* = 3). In this group, the overall response rate (ORR) was 27.7% and the disease control rate (DCR) was 44.4% (Table 3).

**Figure 2:**
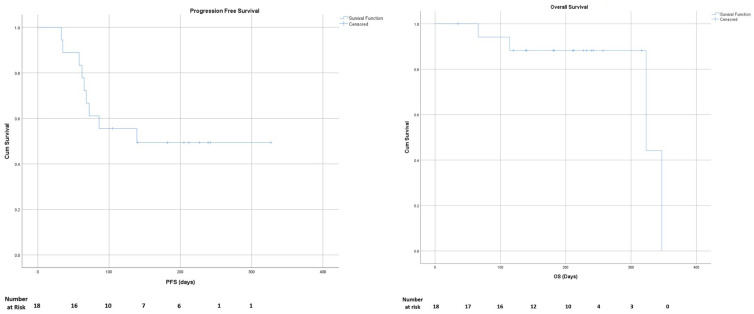
Kaplan–Meier curves for progression-free survival (PFS) and overall survival (OS) in patients with previously treated small cell lung cancer receiving Tarlatamab.

Early deaths during treatment were attributed to disease progression and were not considered treatment-related. The median progression-free survival (PFS) was 139 days. Median overall survival (OS) was 323 days (95% CI, 31–614). On multivariable Cox proportional hazards regression analysis, no variables were independently associated with outcomes (Figure 2).

Among the 18 patients with documented disease courses, six received additional systemic therapy after disease progression on Tarlatamab, including lurbinectedin (*n* = 4), carboplatin, etoposide, and atezolizumab (*n* = 1), and irinotecan with palliative radiation (*n* = 1).

## 4. Discussion

Tarlatamab—a bispecific T-cell engager targeting DLL3—has emerged as a novel therapeutic advance in relapsed SCLC, a malignancy historically characterized by rapid and aggressive progression with limited benefits from later-line systemic therapies [20,21]. In this context, our study supports the growing body of real-world evidence outside the clinical trial setting regarding the feasibility and clinical activity of Tarlatamab.

The clinical characteristics of this cohort reflect the variability inherent to real-world practice. Compared to participants in the DeLLphi trials, our patients were older (median age 72 vs. 64). Although the median number of prior lines of therapy was lower (1 vs. 2 in DeLLphi), 43% had received two or more prior lines, highlighting the broader treatment spectrum encountered outside of clinical trials. In addition, while DeLLphi-301 had majority male patients from Asia and Europe with ECOG of 0 to 1, our cohort included a high proportion of Hispanic patients (61%), reflecting the demographics of the population served by our institution. While the DeLLphi trials permitted patients with stable brain metastases, the incidence in our group was notably higher, with 61% presenting brain metastases within eight weeks prior to initiating Tarlatamab. Early disease-related deaths, the high prevalence of brain metastases at baseline, and the clinical vulnerability not fully captured by baseline ECOG performance status reflect a more advanced and heterogeneous population at treatment initiation, which likely contributed to shorter treatment duration and survival outcomes compared to those reported in clinical trials.

T-cell-redirecting therapies are commonly associated with immune-mediated adverse events, particularly CRS and ICANS [31]. Compared to landmark clinical trials, where CRS and ICANS were frequent but typically low-grade, we observed a lower overall incidence, with all cases limited to grade 1 or 2. These events occurred early, mainly on C1D1 and C1D8 during inpatient monitoring, and were managed with supportive care, including corticosteroids. The rapid onset and resolution of CRS and ICANS support the value of step-up dosing, early inpatient monitoring, and standardized protocols. Despite a high rate of brain metastases, no increase in neurotoxicity was observed. Most patients with CNS metastases had received prior local therapy, including stereotactic radiosurgery or whole-brain radiation, which should be considered when interpreting the absence of increased neurotoxicity in this cohort. While not designed to assess CNS involvement and ICANS, our findings support the feasibility of Tarlatamab in patients with stable or treated brain metastases.

Antitumor activity in our cohort differed from that reported in landmark clinical trials and prior real-world studies. In our series, partial response was observed in 27.7% of evaluable patients, which is lower than the response rates reported in DeLLphi-301 and DeLLphi-304 (40% and 35%, respectively) and in other real-world cohorts (42.9%) [22]. The high prevalence of baseline CNS metastases reflects the advanced and clinically complex population treated outside of clinical trials and may have influenced overall response rates, highlighting the challenges of response assessment in routine practice. Despite this, the observed DCR of 44.4% and median OS of 323 days signal an antitumor activity broadly consistent with previously reported outcomes, and support the feasibility of using Tarlatamab in the real-world practice.

This study has several limitations. It was a retrospective, single-center analysis with a limited sample size, which may restrict generalizability. Survival analyses were affected by censoring related to ongoing treatment at the time of data cutoff and incomplete longitudinal follow-up inherent to real-world cohorts. In addition, a subset of patients was not evaluable for radiographic response due to early clinical deterioration, transfer of care, or loss to follow-up. Given the heterogeneity of this real-world cohort and the presence of early clinical deterioration in some patients, survival outcomes should be interpreted with caution, as these factors may influence PFS and OS estimates in a cohort of this size. These limitations, along with the small sample size and exploratory intention of survival outcomes preclude formal comparative analyses and limit definitive conclusions regarding PFS and OS. However, this represents one of the earliest real-world evidence studies evaluating the impact of Tarlatamab in patients with small cell lung cancer. Notably, the cohort was characterized by a high disease burden, including a substantial proportion of patients with central nervous system metastases, reflecting a population often excluded from clinical trials. In addition, our study highlights outcomes in a predominantly Hispanic patient population, which remains underrepresented in prospective studies.

As Tarlatamab transitions into outpatient use and expands into community and remote practice settings, additional real-world evidence will be essential to better define its safety, effectiveness, and optimal utilization across diverse and high-risk patient populations.

## 5. Conclusions

Overall, Tarlatamab demonstrates feasibility and clinical activity in real-world practice with a manageable safety profile, though outcomes differ from trials due to patient heterogeneity. Overall, despite a heavily pre-treated population, we are able to see around 38% patients having one-year survival, which in an SCLC setting shows positive gains. Step-up dosing and standardized monitoring protocols remain valuable for managing immune-mediated toxicities. Due to an encouraging safety profile in a real-world population, truncated inpatient observation and remote monitoring protocols should be explored.

## Figures and Tables

**Table 1:** Baseline patient characteristics.

Baseline Patient Characteristics	
Age, years (median, IQR)	72 (56–87)
Sex	
Male	14 (61%)
Female	9 (39%)
Race/Ethnicity	
Hispanic	14 (61%)
Non-Hispanic White	6 (26%)
Black	3 (13%)
Smoking history	
Former	19 (83%)
Current	4 (17%)
ECOG performance status	
0	3 (13%)
1	19 (83%)
2	1 (4%)
Charlson Comorbidity Index (mean ± SD)	9 (±2.1)
Stage at initial diagnosis	
Limited	10 (43%)
Extensive	13 (57%)
Brain metastases at diagnosis	
Yes	3 (13%)
No	20 (87%)
Brain metastases prior to Tarlatamab	
Yes	14 (61%)
No	9 (39%)
Liver metastases at diagnosis	
Yes	3 (13%)
No	20 (87%)
Number of prior lines of therapy before receiving Tarlatamab	
1	13 (57%)
2	5 (22%)
3	4 (17%)
4	1 (4%)

**Table 2:** Adverse events summary (Cycle 1 Day 1 and Cycle 1 Day 8).

Adverse Event	C1D1	C1D8
CRS all grades—no. (%)	4 (17.4)	3 (13.0)
Grade 1	2	2
Grade 2	2	1
Grade ≥ 3	0	0
Received dexamethasone—no. (%)	3 (13.0)	2 (8.7)
Median time of onset—hours (IQR)	7.5 (5.8–11.0)	17.0 (11.5–44.5)
Median time to resolution—hours (IQR)	8.5 (1.8–27.8)	7.0 (6.0–7.0)
ICANS all grades	3 (13.0)	3 (13.0)
Grade 1	1	2
Grade 2	2	1
Grade ≥ 3	0	0
Received dexamethasone—no. (%)	2 (8.7)	2 (8.7)

**Table 3:** Disease outcomes in patients with radiographically evaluable disease after at least one cycle of Tarlatamab.

Disease Outcomes (From n = 18)	
Best Overall Response	n (%)
Complete Response	0 (0)
Partial Response	5 (27.7)
Stable Disease	3 (16.7)
Progression of Disease	10 (55.6)
Objective Response Rate (ORR)	5 (27.7)
Disease Control Rate (DCR)	8 (44.4)
Death before first evaluation scan	5

## Data Availability

The de-identified data presented in this study are available on request from the corresponding author due to patient confidentiality.

## Abbreviations

The following abbreviations are used in this manuscript:

SCLC	Small Cell Lung Cancer
ES-SCLC	Extensive-Stage Small Cell Lung Cancer
LS-SCLC	Limited-Stage Small Cell Lung Cancer
PFS	Progression-Free Survival
OS	Overall Survival
CRS	Cytokine Release Syndrome
ICANS	Immune Effector Cell-Associated Neurotoxicity Syndrome
ECOG	Eastern Cooperative Oncology Group
CNS	Central Nervous System
C1D1	Cycle 1, Day 1
C1D8	Cycle 1, Day 8
IQR	Interquartile Range
FDA	Food and Drug Administration
DLL3	Delta-like Ligand 3
PD-L1	Programmed Death-Ligan 1
mOS	Median Overall Survival
IRB	Internal Review Board
